# Feasibility and application of a retronasal aroma-trapping device to study in vivo aroma release during the consumption of model wine-derived beverages

**DOI:** 10.1002/fsn3.111

**Published:** 2014-04-07

**Authors:** Carolina Muñoz-González, Juan José Rodríguez-Bencomo, Maria Victoria Moreno-Arribas, Maria Ángeles Pozo-Bayón

**Affiliations:** Instituto de Investigación en Ciencias de la Alimentación (CIAL) (CSIC-UAM)C/Nicolás Cabrera, 9, 28049, Madrid, Spain

**Keywords:** Drinking, ethanol, in vivo aroma release, retronasal aroma-trapping device, sugar, wine-derived beverages

## Abstract

New types of wine-derived beverages are now in the market. However, little is known about the impact of ingredient formulation on aroma release during consumption, which is directly linked to consumer preferences and liking. In this study, the optimization and validation of a retronasal aroma-trapping device (RATD) for the in vivo monitoring of aroma release was carried out. This device was applied to assess the impact of two main ingredients (sugar and ethanol) in these types of beverages on in vivo aroma release. Two aroma-trapping materials (Lichrolut and Tenax) were firstly assayed. Tenax provided higher recovery and lower intra- and inter-trap variability. In in vivo conditions, RATD provided an adequate linear range (*R*^2^ > 0.91) between 0 and 50 mg L^−1^ of aroma compounds. Differences in the total aroma release were observed in equally trained panelists. It was proven that the addition of sugar (up to 150 mg kg^−1^) did not have effect on aroma release, while ethanol (up to 40 mg L^−1^) enhanced the aroma release during drinking. The RATD is a useful tool to collect real in vivo data to extract reliable conclusions about the effect of beverage components on aroma release during consumption. The concentration of ethanol should be taken into consideration for the formulation of wine-derived beverages.

## Introduction

The increasing interest of consumers in light, fruity, and low alcohol beverages has caught the attention of the wine industry, which has found in these demands an interesting source of diversification in new types of wine-based products (healthier products with low alcohol content, with added sweeteners, mixed with fruit juices, etc.). Therefore, in the coming years, one of the main challenges for the wine sector will be focused on promoting and diversifying their production.

Aroma is one of the most outstanding characteristics determining food preferences and consumption patterns. Understanding the behavior of aroma molecules in beverages during consumption is necessary for the development of new drinks, which should still taste as good as the reference products. In addition, when producing high -quality beverages, it will be important to determine whether the change in one or several ingredients in the formulation of the product, could affect the aroma release pattern and, therefore, the sensory characteristics of the product.

Different works have shown the impact of wine matrix composition on aroma release in static conditions (Dufour and Bayonove 1999a,b; Dufour and Sauvaitre 2000; Aznar et al. 2004; Pozo Bayón and Reineccius 2009; Robinson et al. 2009; Saenz-Navajas et al. 2010; Munoz-Gonzalez et al. 2011; Rodriguez-Bencomo et al. 2011). Although these types of studies have been very valuable in determining specific interactions between aroma compounds and wine macrocomponents, they were not performed in real consumption situations (drinking conditions). Therefore, aspects of the aroma analysis during the drinking process, which includes the effect of release and transport of the aroma compounds to the olfactory epithelium and other physiological aspects accounted for during drinking (swallowing, breathing, interaction with saliva, adsorption with mucus, etc.), which have been shown to have an outstanding effect on aroma release (Buettner et al. 2001; Weel et al. 2003, 2004; Genovese et al. 2009; Deleris et al. 2011; Smyth and Cozzoino 2013) have not been considered in previous studies.

Clark and collaborators (Clark et al. 2011) have recently shown the enhancement effect of ethanol on aroma release when using in vivo (API-MS) monitoring of aroma compounds during the consumption of flavored model beers, which is in disagreement with the retention effect determined for ethanol in previous studies performed in static conditions (Escalona et al. 1999; Rodriguez-Bencomo et al. 2002; Aznar et al. 2004; Aprea et al. 2007). This fact underlines the necessity for in vivo studies to determine the real influence of beverage composition on aroma release during consumption.

To monitor aroma release during drinking, different approaches can be used, mainly based on the online monitoring of aroma release by using mass spectrometric techniques (API-MS, PTR-MS) (Lindinger et al. 1998; Taylor and R.S.L. 2000) or the off-line monitoring by trapping the exhaled breath after swallowing (by the nose or mouth) onto adsorbent polymers (Delahunty et al. 1996; Buettner and Schieberle 2000; Lasekan et al. 2009). The online monitoring of aroma release by API-MS and PTR-MS has been proven as a sensitive and very valuable tool allowing the real-time monitoring of aroma compounds during eating, permitting the collection of valuable data to compare with the sensory analysis of the same product (Munoz-Gonzalez et al. 2011; Deleris et al. 2013). However, some constraints of this approach are the difficulties in the identification of aroma compounds with the same nominal mass (isobaric compounds), the assignment of fragments of the compound of interest produced during the ionization process, or the identification of aroma compounds when analyzing real food samples with complex aroma mixtures (Munoz-Gonzalez et al. 2011; Poinot et al. 2013). Moreover, the sophistication and high price of the required instrumentation for this type of analysis could be considered as an additional drawback. On the other hand, the use of trapping polymers for *in breath* analysis does not provide the temporal profile of aroma release, therefore, making the interpretation of the sensory results more difficult. Nonetheless, this technique allows the precise identification of the compound of interest and the possibility of concentrating the breath extract increasing its sensitivity (Buettner and Schieberle 2000). In addition, the relatively low cost of this methodology facilitates its implementation in any laboratory.

Therefore, the objectives of this study, were first, to evaluate the feasibility of a retronasal aroma-trapping device (RATD) to evaluate aroma release during the consumption of wine-derived beverages, and second, to apply this methodology to study the influence of two main ingredients (sugar and ethanol) typically used in the formulation of these types of beverages. For the first part of the study, in vitro dynamic headspace conditions (purge and trap) were selected to compare the performance of two types of adsorbents to be used in the RATD, while the validation of the RATD conditions to study aroma release from model wine beverages and its application to evaluate the effect of beverage formulation was performed in real in vivo conditions during drinking.

## Material and Methods

### Model wine-based beverages

For the in vitro dynamic headspace experiments (purge and trap), a model wine made up of ethanol (120 mL L^−1^), Milli Q water, and 3.5 g L^−1^ of tartaric acid was prepared. The pH was adjusted to 3.5 with NaOH (4 mol L^−1^). Aromatization was performed with a mixture of six aroma compounds representative of the wine volatile profile (ethyl hexanoate, *β*-ionone, linalool, guaicol, *β*-phenylethanol, and isoamyl acetate), all of them characterized for having a wide range of physicochemical properties (Table 1). The aroma mixture was prepared in absolute ethanol and added to the wine making a final concentration of 1 mg L^−1^, except for *β*-phenylethanol and guaiacol that were added at concentrations of 15 mg L^−1^ and 4 mg L^−1^, respectively. All the solvents and reactants were purchased from Panreac Química S.A. (Barcelona, Spain).

**Table 1:** Physicochemical properties of the aroma compounds employed in this study

Compound	CAS number	MW (g mol^−1^)	BP (°C)	log *P*^1^
Ethyl hexanoate	123-66-0	144	167	2.83
*β*-Ionone	8013-90-9	192	262	4.42
Linalool	78-70-6	152	204	3.38
Guaiacol	90-05-1	124	211	1.34
*β*-phenylethanol	60-12-8	122	224	1.57
Isoamyl acetate	123-92-2	130	134	2.26

1 log *P* = log of the water partition coefficient estimated from molecular modeling software EPI Suit (U.S. EPA 2000–2007).

For the in vivo aroma release experiments, different low alcohol model wine beverages were prepared. To do so, a hydroalcoholic solution composed of ethanol (5 mL L^−1^), Milli Q water, and 3.5 g of tartaric acid were used. The pH was adjusted to 3.5 by using 3.5 g of citric acid. To make a pleasant beverage for the assessors, only an aroma mixture composed of ethyl hexanoate, isoamyl acetate, and linalool, all at the same concentration (25 mg L^−1^) was employed to aromatize the wines. This model wine beverage was coded as MWB-1. In addition, three others types of model wine beverages were produced by varying the content of ethanol and/or sucrose: MWB-2 was prepared like MWB-1 but adding 15 g L^−1^ of sucrose; MWB-3 was prepared with neither alcohol nor sucrose, and MWB-4 was prepared with sucrose (15 g L^−1^) but without ethanol. Table 2 details the composition of the four model wine beverages. All the solvents and reactants employed for these model wines were food grade and were purchased from Panreac Química S.A.

**Table 2:** Formulation of the model wine-derived beverages

	Model wine-derived beverages			
	
Composition	MWB-1	MWB-2	MWB-3	MWB-4
Aroma mixture^1^	+	+	+	+
Tartaric acid (3.5 g L^−1^)	+	+	+	+
Citric acid (3.5 g L^−1^)	+	+	+	+
Ethanol (5 mL L^−1^)	+	+	−	−
Sucrose (15 g kg^−1^)	−	+	−	+

1 Aroma mixture constituted by isoamyl acetate, ethyl hexanoate, and linalool at the same concentration (25 mg/L). Symbols + and − denote presence or absence of a specific ingredient.

### Dynamic headspace-GCMS analysis

To select the most appropriate adsorbing material to be used in further experiments with the RATD, dynamic headspace sampling conditions (purge and trap) were selected to better approach the dynamic situation accounted for during the drinking process. Preliminary experiments were performed in order to optimize the extraction conditions. In the end, a volume of 100 mL of model wine was placed in a special purge flask (250 mL volume). The sample vessel headspace was flushed with purified nitrogen gas (100 mL min^−1^) during 4 min at 35°C and the purged volatiles were trapped in the selected adsorbent material.

For the trapping material, two different polymers were essayed. The traps were made in the laboratory by using 3 mL empty plastic cartridges (Agilent Technologies, Palo Alto, CA) filled with 100 mg of Tenax TA 60/80 (Sigma-Aldrich, Steinheim, Germany) or Lichrolut EN (Darmstadt, Germany). The adsorbent material was confined between two polyethylene frits (Supelco, Bellefonte, PA). The volatile compounds trapped on the polymers were extracted with 6 mL (3 mL, twice) of a hexane: diethyl ether (1:1) solution through the Tenax trap or dichloromethane in the case of the Lichrolut. A quantity of 30 *μ*L of an internal standard (3-octanol, 25 mg L^−1^) (Sigma-Aldrich) was added to the extract, which was further concentrated under N_2_ stream to a final volume of 200 *μ*L.

Before and after use, the traps were conditioned using 6 mL of the above described organic mixtures and dried under vacuum.

The concentrated extract (2 *μ*L) was injected in splitless mode in the injector port of a Gas Chromatograph Agilent 6890N coupled to a quadrupole Mass Detector Agilent 5973. The injection temperature was set at 270°C. Volatile compounds were separated on a Supra-Wax polar capillary column (60 m × 0.25 mm i.d. × 0.50 *μ*m film thickness) from Konik (Barcelona, Spain). Helium was used as the carrier gas at a flow rate of 1 mL min^−1^. The oven temperature was initially held at 50°C for 2 min, then increased at 8°C min^−1^ to 240°C and held for 15 min.

For the MS system (Agilent 5973N), the temperature readings of the transfer line, quadrupole, and ion source were 270, 150, and 230°C, respectively. Electron impact mass spectra were recorded at 70 eV ionization voltages and the ionization current was 10 *μ*A. The acquisitions were performed in Scan (from 35 to 350 amu) and SIM modes. The identification of compounds was based on the comparison of retention times and mass spectra. The mass spectra were compared with those from NIST 2.0 database. Relative peak areas (RPAs) were obtained by calculating the relative peak area in relation to that of the internal standard. Response factors (RFs) in the MS were calculated by injecting increased concentrations (from 1 to 20 mg L^−1^) of a mixture of the five aroma compounds (all at the same concentration) using the same chromatographic conditions described above. The calculated RFs were 12,319, 12,024, 3849, 10,956, 4740, and 27,726 for isoamyl acetate, ethyl hexanoate, linalool, guaiacol, *β*-phenyl ethanol and *β*-ionone, respectively.

### In vivo aroma trapping using RATD-GCMS analysis

**Figure 1:**
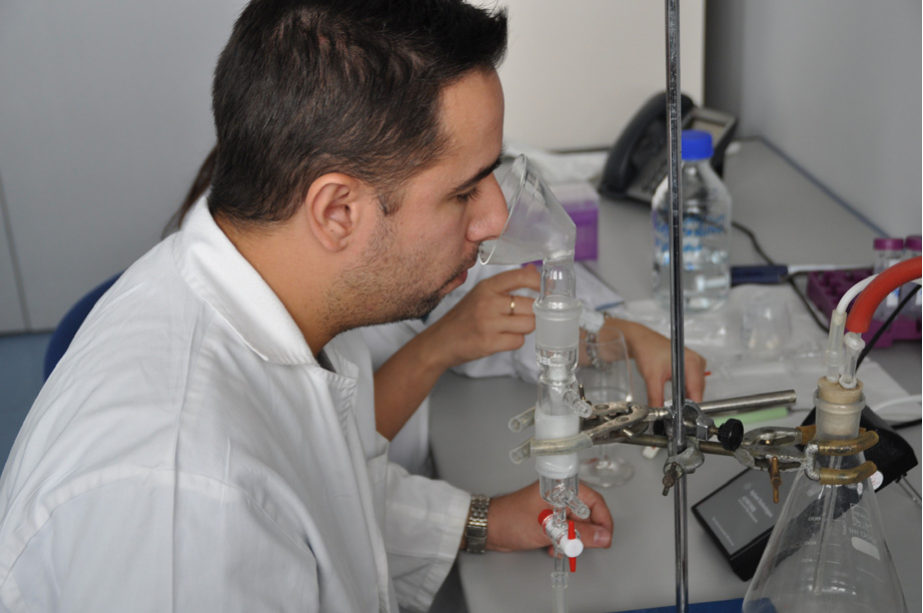
Analysis of retronasal aroma release during the consumption of a wine-derived beverage by using the RATD.

A tailor-made retronasal trapping device (RATD) was employed to trap the exhaled breath of the assessors during drinking. This glass device (Pobel, Madrid, Spain) allowed the trapping of exhaled breath during beverage consumption into a polymeric trap thanks to a glass nosepiece coupled to a hollow tube in which the trap was fitted. A vacuum pump connected to a rotameter allowed a steady flow through the trap. A flowmeter allowed us to know the exact flow through the trap. Figure 1 shows a picture of this device.

Three volunteers (two men and one woman) between 26–34 years previously trained in the retronasal aroma trapping procedure participated in this study. They were instructed not to eat, drink or smoke for 2 h before the experiments. They had no known illnesses and had self-reported normal olfactory and gustatory functions. Before each experiment, the assessors had to clean their mouths and rinse with a bicarbonate solution. The monitorization of the oral cavity of the panelists for the four compounds of interest was performed before each analysis.

The consumption procedure consisted of two steps. In the first one, 20 mL of the model wine beverage was provided to the panelists using a plastic syringe. The sample was kept in the mouth for 20 sec while the assessor had their lips closed in order to favor the equilibration of the aroma compounds within the oral cavity (Buettner and Schieberle 2000). During this time no trapping was performed. After this time, the assessor had to swallow and breathe naturally using the nose through the glass nosepiece for another 20 sec. During these 20 sec, volatiles contained in the breath were trapped into the selected trap. The procedure was repeated until they had consumed 100 mL of the model wine-based beverages. The same trap was used for a single experiment (corresponding to the trapping of the expiration breath of 100 mL of model wine-based beverage). The experiments were carried out in duplicate by using two different traps.

The aroma compounds from the expiration breath trapped onto the trap were desorbed with 6 mL of a hexane/diethyl ether solution (1:1). A volume of 30 *μ*L of an internal standard (3-octanol) was added, and the sample was concentrated using a nitrogen stream to a final volume of 200 *μ*L and analyzed in the GC-MS. A volume of 8 *μ*L of the concentrated breathe extract was injected in a cool injection system unit (CIS) (Gerstel, Mülheim an der Ruhr, Germany) in the solvent vent mode. These conditions were previously optimized and were: vent time: 0.26 min, vent flow: 80 mL min^−1^, injection speed: 0.5 *μ*L sec^−1^, injection temperature ranged from −80°C to 270°C with a 12°C sec^−1^ ramp. The variability in the repeatability of the injection mode in these conditions was <5% for the aroma compounds employed in this study. The rest of the analysis was carried out using the same GC-MS conditions described in the section above. RPAs (peak area compound/peak area internal standard) were used to express total aroma release during the in vivo analysis.

### Statistical analysis

One-way analysis of variance (ANOVA) was used to determine the significant effect of the trapping polymer on the recovery of aroma compounds and to determine the inter-individual effect of the panelists on aroma release performance. Two-way ANOVA was employed to find out the effect of sugar and ethanol on the in vivo aroma release during the consumption of the wine-based beverages. Least significant difference (LSD) test was used for mean comparison. Linear regression was employed to establish the regression parameters for each aroma compound released after drinking the model wine beverage and the lack of fit test was used to judge the adequacy of the linear models. The STATISTICA program for Windows version 7.1 was used for data processing (StatSoft, Inc., 2005, http://www.statsoft.com).

## Results and Discussion

### Selection of the most suitable aroma trapping polymer for the in vivo aroma release experiments

**Figure 2:**
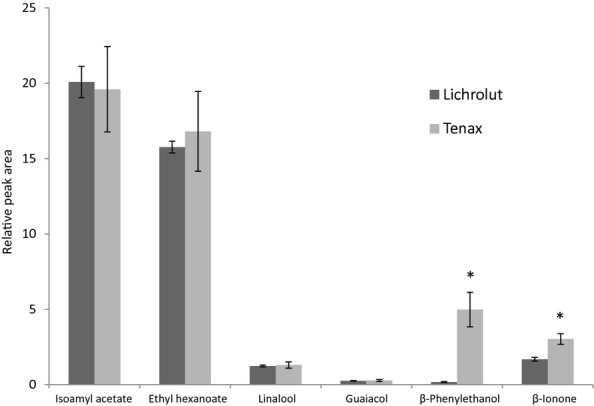
Comparison of the extraction performance (relative peak areas) of the two polymeric traps (Lichrolut and Tenax) employed for the extraction of aroma compounds in a model wine beverage using dynamic headspace analysis. Asterisks denote significant differences among samples (P < 0.05).

Most of the trapping devices described in the literature to monitor in vivo or in vitro food aroma release are based on the use of Tenax (Buettner and Schieberle 2000; Margomenou et al. 2000; Lasekan et al. 2009) as the adsorbent material to entrap the volatile compounds contained in the so-called exhalation breath through the mouth or through the nose. In the case of wine aroma analysis, other entrapping polymers (such as Lichrolut) are often used and it has been proven to give very good performance for the isolation of wine volatiles (Lopez et al. 2002; Andujar-Ortiz et al. 2009). Therefore, the first step in the study was to select the most suitable polymer, among Tenax and Lichrolut to be used in the RATD for the in vivo aroma release experiments with model wine-derived beverages. In order to test these two types of materials, dynamic headspace analysis was used as experimental approach in trying to mimic, as much as possible, the dynamic working conditions of the RATD during the in vivo aroma analysis, avoiding the use of human subject in this first step of the work, which is linked to some experimental drawbacks (inter-individual differences, fatigue, limited number of experiments, etc.). For this type of analysis, aroma compounds contained in a model wine were flushed with a N_2_ stream and trapped in the corresponding polymer. Figure 2 shows the comparison between both types of traps. As can be seen, both trapping materials provided in general, the same extraction yield for most of the aroma compounds. However, *β*-ionone and *β*-phenylethanol were more significantly recovered using Tenax.

Regarding the extraction yield of different types of aroma compounds by using the same adsorbent polymer, it is important to consider that not only the affinity of the compounds for the adsorbent material but also their RFs in the MS (see Dynamic headspace-GCMS analysis in Material and Methods for RFs values) can also affect. In this sense, the esters isoamyl acetate and ethyl hexanoate with the highest RFs also showed the greatest extraction yields independently of the employed polymer. On the contrary, guaiacol showed the lowest recovery no matter the polymer employed for the trapping. This was not because of its poor signal in the MS detector, since other compounds with lower RFs than guaiacol (e.g., linalool, *β*-phenylethanol) were, however, more recovered. Previous works have also shown a low recovery of this compound during the SPE analysis of wine volatiles using Lichrolut (Lopez et al. 2002; Andujar-Ortiz et al. 2009). Similarly, very less *β*-ionone was recovered with either of the two traps, while it exhibited quite high RFs in the MS. In general, the aroma compounds with the lowest log *P* values (Table 1), like guaicol, were the least recovered. This contrasts with results from Aznar et al. (2004) who showed a decrease in the headspace of ethanolic solutions with an increase in the log *P* value until log *P* = 3. This disagreement could be due to the different methodology employed in the above-mentioned study (static headspace) compared to this one (dynamic headspace). In dynamic conditions, Tsachaki et al. (2005) did not find a clear relationship between log *P* and headspace release, which they attributed to the surface active properties of ethanol, which is involved in the so-called Marangoni effect.

The *inter*- and *intra*-trap variations during the extraction using both types of polymers were also determined (Table 3). The intra-trap variation (*n* = 5) was lower than 10% for most of the compounds using both types of trapping materials; however, it was higher (14.7%) for β-phenylethanol by using Lichrolut and for guaiacol (13.5%) using Tenax. These two compounds also had the lowest log *P* values, as mentioned earlier.

**Table 3:** Intra- and Inter-trap variation using Tenax and Lichrolut polymers during the dynamic headspace analysis (purge and trap) of the model wines

	*Intra*-Trap RSD (%)		*Inter*-Trap RSD (%)	
		
	Lichrolut	Tenax	Lichrolut	Tenax
Isoamyl acetate	5.19	8.75	19.22	7.28
Ethyl hexanoate	2.49	8.83	22.00	8.24
Linalool	4.95	8.05	22.21	5.53
Guaiacol	9.54	13.54	7.47	14.58
β-Phenylethanol	14.70	3.86	13.27	6.30
β-Ionone	7.32	7.99	29.59	4.01

RSD, Relative standard deviation (%); *n* = 5 in both experiments.

Many more differences between the two types of polymers were found when comparing the inter-trap variability (*n* = 5). Herein, Lichrolut clearly showed the highest variation, while Tenax trap in general kept, very similar values to those calculated for the intra-trap variation (<10%) for all the aroma compounds except guaiacol. Therefore, taking into consideration the good recovery for most of the volatile compounds of interest and the lower inter- and intra-trap variability, we decided to use Tenax for the in vivo retronasal trapping device.

### Analytical performance of the retronasal aroma trapping device

Once the trapping material was selected, the analytical performance of the RATD in real experimental conditions (drinking conditions) using human assessors was tested. For these experiments, the model wine beverage MWB-1 was used. To improve the acceptability and pleasantness of the beverages for the assessors, only an aroma mixture composed of three aroma compounds (ethyl hexanoate, isoamyl acetate, and linalool) at the same concentration (25 mg L^−1^) was used to aromatize the wine-based beverages for all the in vivo experiments.

#### Dynamic linear range of the RATD

**Figure 3:**
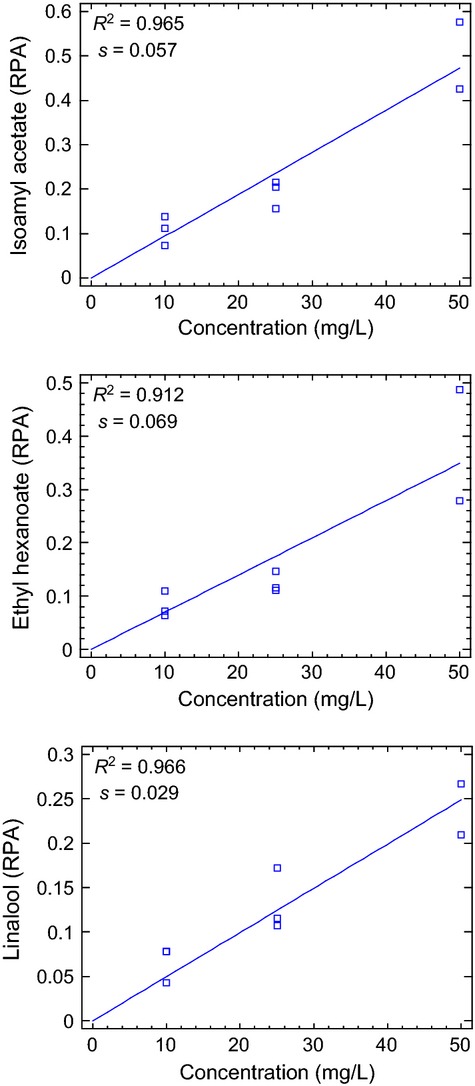
Regression models calculated for the three aroma compounds after the consumption of a model wine beverage with different aroma concentrations by using the RATD-GCMS analysis. *P* values for the calculated modes were: 0.00001, 0.0001 and 0.00001 for isoamyl acetate, ethyl hexanoate and linalool respectively.

The dynamic linear range of the RATD was calculated for the three compounds of interest. To do so, the same beverage (MWB-1) was prepared spiking different concentrations of the mixture of aroma compounds covering a wide range of concentrations (0, 10, 25, and 50 mg L^−1^). Following previous studies (Buettner and Schieberle 2000) and in order to avoid the inter-individual differences, the beverage was consumed by the same panelist following the in vivo aroma release procedure in two different sessions as previously described. The regression models calculated for the compounds of interest are depicted in Figure 3. A lack of fit test was also applied to determine whether the calculated model was adequate for the experimental data. As can be seen, a clear linear relationship between the amount of aroma compounds (RPAs) in the exhaled breath of the individual and the concentration of aroma compounds in the beverages was obtained. The linear models showed determination coefficients higher than 90% for the three essayed compounds: ethyl hexanoate (*R*^2^ = 0.911), isoamyl acetate (*R*^2^ = 0.964), and linalool (*R*^2^ = 0.966) and adequate values of residual standard deviation(s) in the concentration range between 0 and 50 mg L^−1^, showing the adequacy of the RATD to study aroma release in this type of wine samples.

#### Inter-individual differences on aroma release patterns

**Figure 4:**
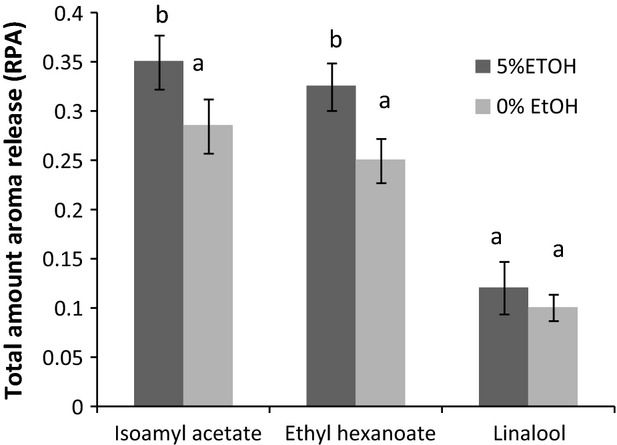
Total aroma release (relative peak area) during the consumption of MWB-1 by three trained assessors determined by RATD-GCMS analysis. Different letters across the different assessors denotes statistical differences (P < 0.05) after the application of the LSD test.

The variability on the total aroma released between panelists equally trained in the same consumption procedure and using the optimized RATD conditions was also determined. Three panelists were instructed to drink the same type of beverage (MWB-1) following the previously described drinking procedure. The graphs showing the aroma release during consumption are presented in Figure 4. As can be seen, in spite of the training, the panelists exhibited significant differences on the total aroma release patterns (expressed as relative peak area) during drinking. Assessor 3 exhibited the highest aroma release for isoamyl acetate and ethyl hexanoate, while assessors 1 and 2 did not show significant differences for the release of isoamyl acetate, and they also slightly differed on the release of ethyl hexanoate. However, no significant differences in the release of linalool were found between the panelists. Inter-individual differences in the aroma release patterns during drinking were previously observed because of the differences in physiological variables (mouth volumes, swallowing, breathing patterns, etc.; Buettner et al. 2001, 2002; Weel et al. 2003, 2004; Deleris et al. 2011). In addition to these physiological factors, these results clearly showed that the type of aroma compound (physicochemical characteristics) also has a significant influence on the aroma release that is in agreement with previous works performed in other food matrices and with other methodologies to monitor aroma release (Saint-Eve et al. 2009; Deleris et al. 2011, 2013).

### Impact of ethanol and sugar on in vivo aroma release during the consumption of model wine beverages

Some works performed in in vitro conditions (static or dynamic headspace conditions) have shown that wine matrix composition might play an important role on the interaction with aroma compounds (Dufour and Bayonove 1999a,b; Dufour and Sauvaitre 2000; Pozo Bayón and Reineccius 2009; Robinson et al. 2009; Rodriguez-Bencomo et al. 2011). Even so, these interactions might affect the sensory characteristics of the wines (Jones et al. 2008; Saenz-Navajas et al. 2010). Therefore, any attempt to formulate any type of wine-based beverage should determine whether the composition might be considered as an important variable when determining aroma release during real drinking conditions. Thus, once the validity of the RATD to determine the aroma released during the consumption of these types of beverages was proven, four model wine beverages were formulated following the recipes previously described differing in two main ingredients; the presence or absence of ethanol and sugar (Table 2). Despite the fact that both ingredients have been described to have a large influence on aroma release from wines in static headspace aroma analysis (Escalona et al. 1999; Rodriguez-Bencomo et al. 2002; Aznar et al. 2004), their influence during the in vivo consumption of wine or wine-based beverages has not been currently explored. To determine solely the effect of matrix composition on aroma release, while avoiding the inter-individual differences previously shown, each of the four model wine beverages (MWB-1, MWB-2, MWB-3, and MWB-4) was consumed by a single assessor in two different sessions following the procedure previously described using the RATD.

Aroma release data were submitted to a two-way factorial ANOVA to determine the effect of the two ingredients. Results from the test did not show a significant effect of adding 15 g L^−1^ of sucrose into the beverage. However, a significant effect of ethanol was shown on the release of isoamyl acetate and ethyl hexanoate. The absence of a significant effect of sucrose on the aroma release is in agreement with the results from Weel et al. (2003), who did not observe differences in aroma release during the consumption of a 10 g L^−1^ sucrose added to a lemon–lime type beverage compared with the reference beverage without the sweetener. In addition, Saint-Eve et al. (2009) did not find a significant influence in the addition of 1 g L^−1^ sucrose on the aroma release during the consumption of mint flavored beverages either. However, other scientific works performed in static conditions, have pointed out some sucrose-flavor physicochemical interactions, although in general, these works were performed with higher sucrose concentrations (20–60 g L^−1^), and have been attributed to a *salting out* effect of sucrose, whereby sucrose interacts with water, increasing the concentration of flavor compounds in the remaining free water (Nahon et al. 1998; Friel et al. 2000; Hansson et al. 2001). Therefore, considering that the concentration of sucrose fit within the concentrations normally used in these beverages (5–15 g L^−1^), it could be concluded that the concentration of sugar does not have a significant effect on aroma release during drinking. Nonetheless, it is important to highlight that this conclusion might not be true for the aroma perception, since perceptual differences are also linked to psychophysical effects (Weel et al. 2003).

**Figure 5:**
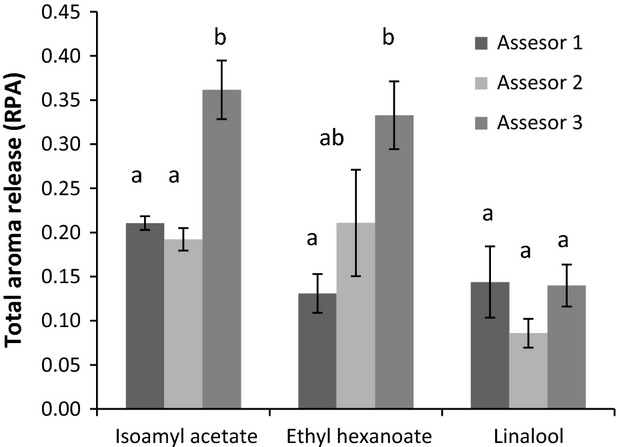
Influence of ethanol on the aroma release during the consumption of model wine beverages using RATD-GCMS analysis. Different letters across the different wine samples denotes statistical differences (P < 0.05) after the application of the LSD test.

Regarding the influence of ethanol on aroma release, an LSD test was applied to the data in order to determine the magnitude of the observed effect. Figure 5 shows these results in which the average aroma release during the consumption of the model wine beverages with and without ethanol (independently of the sugar content, as it did not significantly affect aroma release) are shown. As can be seen, the presence of 5 mL L^−1^ ethanol increased the aroma release during consumption, above 18% for isoamyl acetate and 22% in the case of ethyl hexanoate. For linalool, the average aroma release values were also higher in the model wines with ethanol, although they were not statistically significant. However, these results showed the same trend; an enhancement of aroma release in presence of ethanol. Recently, Clark et al. (2011) also showed by using in vivo-API-MS a similar rise in the release of three targeted aroma compounds during the in vivo consumption of flavored model beers with an increase in the ethanol content from 0 to 4.5 mL L^−1^. Contrarily, most of the studies dealing with the effect of ethanol on aroma release performed in static conditions have shown a reduction in the aroma released into the headspace. This effect has been explained as consequence of the higher solubility of aroma compounds due to an increase in the ethanol concentration (Aznar et al. 2004; Aprea et al. 2007). This fact highlights the idea that static headspace techniques used to monitor aroma release do not provide the same conclusions as the works performed in vivo, independently of the methodology used to monitor aroma release during consumption (online employing API-MS or off-line using the RATD as in this work). Differences between in vitro and in vivo studies might be due to the effect of all the oro-physiological parameters (breathing and swallowing patterns, saliva, mucus, etc.) involved in the in vivo delivery of aroma compounds during drinking.

To explain the enhancement effect of ethanol on aroma release in the in vivo studies, different hypotheses have been proposed (Clark et al. 2011). The first one has been associated with the change that ethanol might induce in surface tension affecting the distribution of the liquid in the mouth and pharynx during consumption, allowing the sample to better spread out and favoring the formation of a larger surface in the pharynx for volatile release. Another effect of ethanol could be linked to its capacity to increase the solubility of aroma compounds in the aqueous coating of the mouth and throat preventing losses and/or increasing the amount of volatile compounds at the gas–liquid interface, which might enhance aroma release. Finally, the so-called Marangoni effect (Hosoi and Bush 2001), could also be involved. In this case, the evaporation of ethanol in the gas–liquid interface of the mouth and throat might create a streaming of new ethanol molecules and volatile compounds to replenish those released, which might increase the amount of aroma released (Tsachaki et al. 2005).

## Conclusions

In summary, the RATD and the consumption procedure optimized in this study allows in a simple, convenient, and precise way, the determination of the impact of matrix components on aroma release during real drinking conditions of model wine-derived beverages. The impact of ethanol, increasing the total amount of aroma release during drinking has been proven, which should be taken into consideration during the formulation of new types of wine-derived beverages; however, the impact of this fact on the sensory characteristics of the beverage should be achieved in future works. In addition, results of this study have shown the importance of collecting real in vivo data to extract truthful conclusions about the effect of beverages components on aroma release during consumption, highlighting the idea that besides its composition, the overall perceived flavor of a food or beverage is mainly impacted by the way in which volatile aroma compounds are released in the mouth and transported to the olfactory receptors in the nose during consumption. However, new experiments involving a higher number of assessors and sensory test should be performed in order to corroborate the effect of these two ingredients on aroma release and their impact on aroma perception.
